# “A perfect society”— Swedish policymakers’ ethical and social views on preconception expanded carrier screening

**DOI:** 10.1007/s12687-018-0389-x

**Published:** 2018-09-26

**Authors:** Amal Matar, Mats G. Hansson, Anna T. Höglund

**Affiliations:** Department of Public Health and Caring Sciences, Center for Research Ethics and Bioethics, Uppsala University, Box 564, SE-751 22 Uppsala, Sweden

**Keywords:** Preconception, Expanded carrier screening, Genetic, Policymakers, Reproductive decision-making, Ethics, Social effects

## Abstract

To improve healthcare policymaking, commentators have recommended the use of evidence, health technology assessment, priority setting, and public engagement in the process of policymaking. Preconception expanded carrier screening, according to the World Health Organization’s definition, is a novel health technology and therefore warrants assessment, part of which involves evaluating ethical and social implications. We examined ten Swedish policymakers’ perspectives on ethical and social aspects of preconception expanded screening through in-depth expert interviewing, using a semi-structured questionnaire. Respondents were affiliated to governmental and non-governmental institutions that directly influence healthcare policymaking in Sweden. The interviews were recorded, transcribed verbatim, and analyzed via inductive thematic analysis method, which generated seven themes and several subthemes. Policymakers harbored concerns regarding the economics, Swedish and international political respects, implementation procedures, and societal effects, which included long-term ones. Moreover, participants detailed the role of public engagement, research, and responsibility in regard to preconception expanded carrier screening implementation. Since this is a qualitative study, with a small non-random sample, the results may not be generalizable to all policymakers in Sweden. However, the results give a profound insight into the process and interpretative knowledge of experts, in the Swedish milieu and the extent of readiness of Sweden to implement a preconception expanded carrier screening program.

## Background

Policymaking has been defined as “authoritative allocation of values” (Easton 1953) where authoritative figures issue directives on behalf of and for a set of people, who are expected to comply with them (Hanney et al. 2003). Health policy refers to both governments’ and non-governments’ strategies, decisions, and undertakings to accomplish particular healthcare goals in a society (Nature.com 2018). It encompasses a wide range of outcomes, for example, national policies, professional medical guidelines, and as thus a variety of persons are engaged in healthcare policymaking, such as politicians, physicians, and managers (Hanney et al. 2003).

In recent years, healthcare systems in the developed countries are encountering challenges with regard to quality of service, equitable accessibility of care, locating adequate funding, growing demand for transparency, and maintainability (Kenny and Joffres 2008). As a result, many measures have been proposed and/or employed to resolve the aforementioned problems, namely, incorporating ethics in health policymaking (Kenny and Giacomini 2005), introducing priority setting procedures (Carlsson 2004; Kenny and Joffres 2008), conducting health technology assessment (HTA) for new health technologies (Carlsson 2004), implementing evidence-based policymaking, and engaging the public in the process of health policymaking (Hanney et al. 2003).

## Preconception expanded carrier screening

The earliest genetic screening performed was neonatal screening for phenylketonuria (PKU) in the USA, followed by screening African Americans for sickle cell trait in the1970s (Lewis 2008). The discovery of fetal genetic material in amniotic fluid, in 1970, paved the way for prenatal genetic screening of pregnant women using amniocentesis (Press 2008). In addition, carrier screening programs for targeted groups such as Tay-Sachs program for Ashkenazi Jews proved successful (Zlotogora 2009). Nevertheless, since its inception, genetic screening raised many ethical and social issues, such as stigmatization, infringement on privacy, and reproductive autonomy and equality of access (Lappé et al. 1972).

Targeted carrier screening of high-risk groups or families is distinct from preconception expanded carrier screening (ECS), which is a form of a new health technology (HT), where a test panel screens for several autosomal and X-linked recessive traits simultaneously. The panel is to be offered to potential parents without prior risk, who are planning a pregnancy. If both parents test positive for a certain monogenic recessive trait, they have a 25% chance of having a child with the disease with each pregnancy (Henneman et al. 2016).

Health technology (HT), as defined by World Health Organization (WHO), is “the application of organized knowledge and skills in the form of devices, medicines, vaccines, procedures and systems developed to solve a health problem and improve quality of lives” (World Health Organization 2018).

By virtue of this definition, preconception ECS can be regarded as a new technology warranting HTA. Swedish Council on Technology Assessment in Healthcare and Assessment of Social Services—Statens beredning för medicinsk och social utvärdering (SBU, see later), defined HTA as the systematic assessment of scientific evidence of methods or materials employed in healthcare prevention, diagnosis, and treatment/care. The assessment involves evaluating potential risks and social and ethical consequences as well as costs and effects, while taking in consideration the local and national situation (Swedish Agency for Health Technology Assessment and Assessment of Social Services 2018).

## Healthcare decision-making in Sweden

Sweden is a parliamentary democracy, where the parliament has the legislative power in the country. Swedes select their parliament every 4 years via national elections. The parliament reviews and votes on proposed draft laws by the Swedish government (The Government Offices of Sweden 2014).

The Swedish government is formed of a Prime Minister and 23 ministers, in which there are at the time of this study, three ministers in charge of Ministry of Health and Social Affairs, namely Minister of Social Security; Minister for Children, Elderly and Gender Equality; and lastly Minister of Health Care, Public Health and Sport (The Government Offices of Sweden 2014). The parliament and the ministers constitute the national level of administration whose role is to lay down the political agenda and institute values and guidelines for Swedish healthcare (Clinical Studies Sweden 2017). Healthcare provision in Sweden is publicly funded by taxpayers’ money (Carlsson 2004).

At a local level, there are 21 Swedish county councils and 290 municipalities, which are self-governing entities and its officials are elected every 4 years by voters in their respective locations. County councils are in charge of delivery and funding healthcare services to county’s residents while municipalities take care of the elderly, citizens with disability, convalescent care, and healthcare in schools. Both abide by the agenda set by the national government (Carlsson 2004; Swedish Research Council 2017), yet they retain much freedom in determining how to organize and manage services and expenditures (Carlsson 2004).

The Ministry of Health and Social Affairs has several national boards reporting to it. They include but are not limited to SBU (Statens beredning för medicinsk och social utvärdering—Swedish Council on Technology Assessment in Healthcare and Assessment of Social Services), Socialstyrelsen (The National Board for Health and Welfare), and SMER (Statens Medicinsk-Etiska Rådet—Swedish Medical Ethics Council).

SBU, which is one of the earliest HTA units to be established worldwide, “is an independent national authority, tasked by the government with assessing health care and social service interventions from a broad perspective, covering medical, economic, ethical and social aspects” (Swedish Agency for Health Technology Assessment and Assessment of Social Services 2018). The National Board for Health and Welfare (Socialstyrelsen) is mandated to assure high-quality and equal healthcare and social care for Swedish citizens by drafting medical guidelines, managing health-related information, and upholding health records registries. The board has several committees that function under its umbrella, one of which is an ethical committee (Socialstyrelsen 2018). Lastly, SMER is an independent body issuing recommendations to the government on ethical issues related to biomedical technologies. On board are representatives of the main eight political parties as well as legal, medical, and bioethical experts. Each expert operates for a 3-year period on SMER (Socialdepartementet 2018).

Apart from governmental institutions, there are ethics committees in non-governmental organizations that act as lobbyists and promote their own views on healthcare issues, such as the Swedish Society of Medicine and the Swedish Medical Association. The Swedish Society of Medicine is an independent professional organization concerned with promoting health, research, ethics, and quality within the healthcare system. The organization gives input in debates and on policy documents and guidelines, funds medical research, and disseminates medical information among healthcare professionals. It also responds to referral and investigations requested by the government (Svenska Läkaresällskapet 2018). The Swedish Medical Association is the union for medical doctors who work in Sweden. Besides negotiating better working conditions for doctors, the Swedish Medical Association is also involved with physicians’ professional matters, such as education and development, leadership, research, and ethics (Sveriges Läkarförbund 2016). Both these organizations do not report to the government but can be requested to give their input on certain healthcare policies (Svenska Läkaresällskapet 2018; Sveriges Läkarförbund 2016).

The European Society of Human Genetics (ESHG) published an article showing evidence of the public’s and the professionals’ receptiveness to use ECS (Henneman et al. 2016; Plantinga et al. 2016). Therefore, it can be expected that ECS may be implemented in some EU countries and thus, the authors were of the opinion it is pertinent to investigate Swedish stakeholders’ views and perspectives on this new HT. In an earlier study, we examined healthcare professionals’ ethical and social standpoints on preconception ECS (Matar et al. 2016); and in this study, we are exploring the point of views and perspectives of Swedish healthcare policymakers, another important stakeholder group in a welfare state, such as Sweden.

## Aims

The main aim of the study is to explore and describe how healthcare policymaking experts perceive ethical and social aspects of preconception ECS as a health technology.

As such, we formulated the following specific research questions:

- How do policymakers decide on new health technologies?
- What do they consider in the evaluation of new health technologies to make them warrant public use?
- What are the ethical and social considerations they keep in mind before they decide on implementing new health technologies, e.g., preconception ECS?

## Methods

### Expert interview

Our research participants are defined as experts; therefore, we have employed an expert interview method as described by Bogner and Menz (2009). We specifically utilized systematic expert interviews, which aim to access systematic and complete information about knowledge and experience of an expert. This category can be differentiated from other forms of expert interviewing, when they are being used as exploratory tools or for theory generation (Bogner and Menz 2009). Systematizing expert interview is aimed to access objective knowledge and specialized information acquired by an expert. In our study, we aspire to obtain experts’ views and opinions on ethical and social aspects of a new HT, namely, preconception ECS.

To select an expert for our study, we depended on a “social representational” line of reasoning in contrast to method-relational approach (Bogner and Menz 2009). By the former, we mean that an expert was selected because s/he has the social components that established him/her as an expert, for instance, his/her position in professional organizations or his/her list of publications. Method relational approach, on the other hand, does not consider this social representation but judges an expert as someone who has the most practical insight into the workings of an organization. Therefore, an expert could belong to a low to middle hierarchy personnel within the organization (Bogner and Menz 2009). In our study, all the interviewees serve in committees that directly affect health policymaking in Sweden.

Our goal was to access experts’ process and interpretative knowledge as defined by Bogner and Menz (2009). They categorized knowledge into technical, process, and interpretative. Process knowledge describes practical experience of the expert, such as procedures and routines, while interpretative knowledge explains experts’ decisions, interpretations, opinions, subjective reasoning, and nuances and so on.

### Participants

Our sample consisted of four female and six male respondents: four physicians, three bioethicists, one legal expert, one political party representative, and one theologian. All the participants were members of committees that can directly influence the healthcare policymaking in Sweden. These committees addressed ethical and social aspects of proposed healthcare procedures either as part of their main operation framework, such as SMER or SBU, or via an ethical subcommittee, for example, Socialstyrelsen, Swedish Medical Association, or Swedish Society of Medicine (Table 1).

**Table 1:** Participants’ demographics

Profession	Gender	Committee
• 4 physicians • 3 bioethicists • 1 legal expert • 1 theologian • 1 political party representative	• 4 females • 6 males	• SMER^1^ • SBU^2^ • Ethical board of Socialstyrelsen^3^ • Swedish Society of Medicine • Swedish Medical Association

^1^Statens Medicinsk-Etiska Rådet—Swedish Medical Ethics Council

^2^Statens beredning för medicinsk och social utvärdering—Swedish Council on Technology Assessment in Healthcare and Assessment of Social Services

^3^The National Board for Health and Welfare

At the time of the interview, the interviewees served or were serving in one or two of the following committees:

- SMER (Statens Medicinsk-Etiska Råd—Swedish Medical Ethics Council)
- SBU (Statens beredning för medicinsk och social utvärdering—Swedish Council on Technology Assessment in Healthcare)
- Ethical board of Socialstyrelsen (The National Board for Health and Welfare)
- Swedish Society of Medicine (Svenska Läkaresällskapet)
- Swedish Medical Association (Sveriges Läkarförbundet)

### Data collection

Data collection for the study started in February and was completed by November, 2017, by the first author. To procure interviewees for the study, we consulted the websites of the aforementioned committees to obtain interviewees’ contact details, after which, we resorted to a snowballing method of recruiting research participants. By the later method, we mean that participants who were interviewed were asked for names and contact details of potential candidates who serve in one or more of the committees aforementioned. We have contacted a total of 30 persons, ten of whom agreed to be part of our study and thus were included.

The interview guide was designed following a thorough literature review and was reviewed and agreed upon by all authors. It was divided into three parts: one part inquired about interviewees’ background and expertise, the second part was dedicated to healthcare decision-making, and the last part of the interview addressed ethical and social issues related to preconception expanded carrier screening (Table 2). All the interviews were conducted in English.

**Table 2:** Interview guide

Section	Questions
Demographics	• Professional background, function as policymaker, description of their role as policymaker • Have you heard of preconception expanded genetic screening? If yes, in what context?
Healthcare decision-making	• What would influence/impact your judgment in assessing preconception ECS? Are there certain ideologies? Values? Interests you would keep in consideration? What are they? • Would you advocate for public engagement in deciding on implementing preconception ECS in Sweden? Why and to what extent? • What type of research do you need to consider in evaluating preconception ECS? • What about economic considerations? In case of situations with limited resources, should preconception ECS to be prioritized? Why?
Preconception ECS	• Can you think of any value conflicts when deciding on preconception ECS? What are these values and what obstacles can you foresee? • From your perspectives, what are the ethical issues to consider when evaluating preconception ECS? • From your perspectives, what are the social issues to consider when evaluating preconception ECS? • From your point of view, what are the positive consequences generated by implementing preconception-expanded carrier screening? For parents, for healthcare system? For society? • What are the potentially negative consequences? For parents, for healthcare system? For society? • What would make Swedish healthcare consider implementation of preconception ECS? What is your stance on that?

The duration of the interviews ranged between 41 and 68 min, where the majority of the interviews (six interviews) lasted more than an hour. The interviews were recorded and transcribed verbatim by a transcription company called Rappa Tag Skrivcenter.

### Analysis

**Fig. 1:**
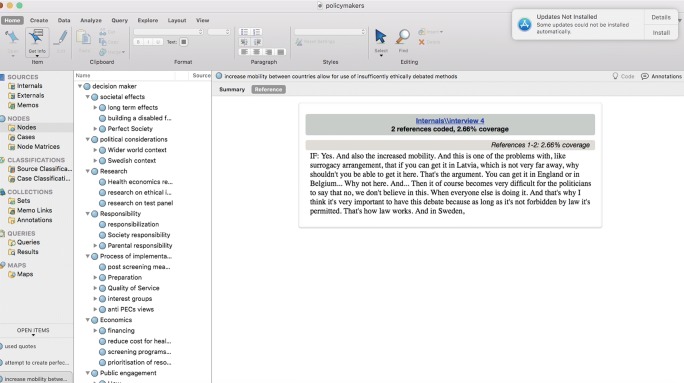
Analysis process by NVivo

All transcripts were revised, then read through once before the initial open coding was performed. The codes were sorted into major themes. Under each theme, the quotes were further examined to identify subthemes. The analysis followed an inductive approach to locate the different themes and subthemes. These steps were performed by the first author, who utilized NVivo 11.4.3 software for the analysis (Fig. 1). We followed thematic analysis method as described by Ryan and Russell Bernard (2003).

During design of the study, data collection, and analysis, elements of credibility, transferability, and dependability of data were given due consideration in order to achieve trustworthiness of the analysis. Credibility means trust in study design and analysis of a qualitative study to best fulfill its aim and answer its research question. This has been achieved, in our study, by selecting members of different genders and professions, who served on different committees both governmental and non-governmental. Moreover, we used expert interviewing as a method to be able to access participants’ systematic knowledge and experience. The themes and subthemes were agreed upon after discussions between authors and a consensus reached (Graneheim and Lundman 2004).

Dependability, the second aspect of trustworthiness, refers to possible inconsistencies occurring as a result of prolonged time of data collection or adjustments made by the investigators when analyzing data (Graneheim and Lundman 2004). The time duration for data collection, in our study, was not long and minimal changes occurred in the interview guide. During the analysis, process adjustments were only made after discussions and consensus between authors.

The last component of trustworthiness is transferability, which denotes the extent the study outcomes can be passed on to similar settings or units. This can be accomplished by accurately describing the setting and frame of reference, research participants’ selection, and attributes as well as the analysis process (Graneheim and Lundman 2004). This has been achieved by detailing aspects of the Swedish political scene in relation to the healthcare system, the functions of different committees from which participants were selected, the reporting of participants’ profession genders, and place of work. We have also described the methods for data collection (expert interviews) and the analysis process (thematic analysis).

### Ethics

According to Swedish Ethical Review Act (SFS 2003: 460), no ethical review is required when a research involves interviewing public figures or figures working on public committees (Ministry of Education and Cultural Affairs 2003). Nevertheless, we abided by the Declaration of Helsinki guidelines’ on human subject research participation (General Assembly of the World Medical Association 2014). This has been discussed in more details later in the text (please refer to compliance to ethical standards).

## Results

The analysis of the transcripts produced seven major themes and several subthemes. The main themes were as follows: economics, political considerations, considerations of implementing preconception ECS, role of public engagement, research, responsibility, and lastly, societal effects (Table 3). Below is the description of the themes and ensuing subthemes.

**Table 3:** Themes and subthemes

	Themes	Subthemes
1.	Economics	• Alternative financing • Prioritization of resources • Reduced cost for healthcare
2.	Political considerations	• International context • Swedish context
3.	Considerations of implementing preconception ECS	• Interests groups • Preparation • Post-screening measures • Quality of service • Anti-preconception ECS views and alternatives
4.	Role of public engagement	• Who? • How? • Why?
5.	Research	• Research on ethical issues • Health economics research • Research in relation to test panel
6.	Responsibility	• Parental responsibility • Societal responsibility • Responsibilization
7.	Societal effects	• A disabled-friendly society • Perfect society • Long-term effects

### Economics

Under the economics theme, interviewees indicated *alternative means of financing preconception ECS*, *considered prioritization of resources in healthcare*, and *how preconception ECS may reduce costs to healthcare*.

Regarding financing, one respondent (theologian) was of the opinion that if preconception ECS “isn’t a hundred percent (approved) to have in the ordinary general healthcare” and the test is of an affordable price “up to 2000 SEK,” then it can be made available commercially.

Indeed “mixed financing” or “partial financing” would be on the rise, where the State pays a basic service for all, and if patients required a more advanced option or service, they would pay it themselves. When asked who would decide on “good enough” quality, the respondent replied “it is decided by Swedish healthcare.”

Almost all respondents emphasized and reiterated the concept of prioritization of resources in relation to preconception ECS. They indicated the importance to evaluate preconception ECS from priority setting point of view before implementation. However, one respondent stated that it is difficult to do so because

we are talking about an individual that might not even be existent yet, so how should we value that, that a certain individual comes into existence compared to not coming into existence. And we have difficulty in measuring what is the effect. Should we only look at the effects on the parent or should we look at the effect on the child too etcetera? (bioethicist 1)

Many of the respondents stated that screening programs take up a lot of resources and that include the cost of screening test and dealing with false positive and negative results, as well as training staff like genetic counselors, laboratory staff, etc. Nevertheless, other respondents indicated that preconception ECS may reduce costs for healthcare system in case it substitutes expensive treatments of diseases, such as cystic fibrosis.

### Political considerations

Political considerations are not confined to *Sweden* only; respondents detailed how the *international context* can affect the healthcare system in Sweden. For instance, parents in Sweden may demand preconception ECS because

you can get it in England or in Belgium... Why not here? Then it, of course, becomes very difficult for the politicians to say to that no, we don’t believe in this, when everyone else is doing it.

The respondent added that increased mobility within the EU could allow implementation of methods that have been “insufficiently ethically debated in Sweden” (legal expert).

However, another respondent believed that countries within the EU “see so differently on these (reproductive screening) matters. The ethics, well, ethical rules, so to speak, differ” (political party representative). Indeed, to one respondent, preconception ECS

cuts across many political ideologies. So it doesn’t matter, here, if you’re a conservative or a libertarian or a socialist, right. So this is about matters that you could be very different inside all the established parties. So you cut across the usual party lines in this way (bioethicist 2).

Within the Swedish context, some respondents kept referring to Swedish healthcare values and legislation when a preconception ECS program is evaluated. Participants signified values, such as human dignity; a non-discriminatory principle where everyone is viewed as equal, solidarity; where the most in need have the highest priority for healthcare attention, cost-effectiveness and priority setting, patient’s integrity and autonomy, and lastly respecting privacy as part of healthcare values.

Despite the existence of these values, a few respondents were of the opinion that some political decisions “are not really scientifically (based)” (physician 4) and

Politicians do all kinds of things against better knowledge from experts, I think they do that all the time. And that is of course because they have a political agenda and they think it pleases voters and they are after that (physician 2).

Thus, “many of the problems in healthcare and medicine has little to do with resources and more with government and leadership,” and this would affect the decision regarding preconception ECS.

One participant questioned the ethical motivation that underpins implementation of preconception ECS in Sweden by stating,

So something with, you know, helping people to make decisions, so giving them information that would help them to make decisions. Well, then it’s a whole ethical problem around whether or not that’s a legitimate aim of healthcare, especially public healthcare. Is this what tax money should be for, and so on (bioethicist 2).

Another respondent (physician 4) recommended that all new technologies including preconception ECS, should undergo “ordnat införande” (orderly introduction), which is a system used in Sweden to introduce new drugs.

### Considerations for implementing preconception ECS

This theme comprises several subthemes, which all relate to matters to heed if/when implementing preconception ECS. The subthemes are as follows: *interest groups, preparation phase*, *quality of service*, *post-screening measures*, and *anti-preconception ECS views*.

According to the majority of interviewees, there are several interest groups which could lobby for or against preconception ECS. These include researchers and research institutions, professional organizations, such as the Swedish Society of Medicine, healthcare institutions, patient organizations, commercial organizations, and even politicians. As this quote indicates:

I guess that there will be increased pressure, not the least from commercial interests. If this will be a success story from a commercial point of view, in the US … It will definitely be a commercial pressure also in Sweden. I guess that some professional interests will come in. I don't know about parent organizations for children with rare disorders. There are such organizations and there is an umbrella organization also for those in Sweden … But one possibility is that they consider that their children’s life is worth enough, so there shouldn’t … introduce methods that will more or less extinguish their type of children. So I don’t know if they will be a pressure organization here (physician 1).

One issue raised is some medical researchers’

known tendency to slide from research (to clinical practice). You introduce clinical methods in a research setting and then the professional can’t let go. So, there are so many examples of very imperfect stuff being implemented year after year after year after year, on the hope that it sometime will be proved to be a good thing. And this is faith, it’s not science (bioethicist 2).

For the preparation phase, a few suggestions were made to avoid potential problems. For example, providers could target a pilot group with an information campaign and perform the program using an intervention ladder approach, which would run in stages (bioethicist 2); ensure sufficient personnel are available to carry out the program (physician1); reflect, and “have all the scientific and ethical discussions about it” first (physician 4), because of the difficulty to “take back a screening program” once implemented since,

it’s such a massive undertaking and a lot of people are engaged, a lot of people have their salaries from the screening program, etcetera, etcetera. And they of course become advocates of their own institutions, the screening institutions (bioethicist 3).

Respondents highlighted the importance of the quality of service in context of preconception ECS because if

it’s a sloppy advice and those that give advice are not very knowledgeable, I mean, it can cause mistrust for all screening procedures, it may cause mistrust in the medical services at large (physician 1).

Quality of service extended to quality of information given to users (bioethicist 2), competence of healthcare personnel and quality of lab facilities (bioethicist 1) quality of informed consent and quality assurance of procedures performed during pretest and posttest. Moreover, developers of the program should identify quality criteria that can be monitored at different phases of the service (physician 1).

A few respondents mentioned post screening measures and what those entailed. Would it involve advice on change of partners, or offering measures such as preimplantation diagnosis (physician 1) or lead to unnecessary or excessive treatments (bioethicist 1)? As this quote explains

you might be at risk and you do not do anything about it, does it do good for this person or does it do bad? And put a healthy person in a state of thinking, I might be ill … So you have to have something to offer as well and something that makes the life better for the person who is involved in the screening, I think … I mean, what happens next? It is not just a knowledge, you have to think about the whole chain and how to meet that and do we have the technique and do we have the possibility to continue. (physician 4)

The last subtheme comprises views opposed to preconception ECS and suggestion of alternatives. According to respondents 1, 6, and 7 (political party representative, bioethicists 2 and 3), the current politics both in terms of political parties as well as involved agencies in Sweden would not approve a preconception ECS program.

So one thing I was saying from my experience, from sort of Swedish policy making in this area, that I would view it very unlikely to have this kind of program being okayed in Sweden, as it is now actually. So I don’t think that involved agencies would okay it, I don’t think that it would get a thumbs up from the Health Technology Assessment Agency (bioethicist 2).

Most of the participants expressed views opposing the implementation of preconception ECS citing various rationales. For instance,

I still think it’s more probably going to be harmful, both to autonomy and actually health, than it’s going to help. It’s going to help some selected individuals, but here you have to look at the entire population. So I think from a public health standpoint this is waste of money. (bioethicist 2)

Suggested alternatives to preconception ECS comprised methods already in use within healthcare system, such as NIPT (non-invasive prenatal test) (physician 3), new methods under trials, such as gene editing techniques (bioethicist 3), develop effective treatments to genetic diseases (political party representative), or manage environmental causes of diseases (legal expert).

### Public engagement

Underneath this theme, respondents detailed *who* should be engaged and *how* it should be achieved and *why* it should be carried out. Few respondents were concerned about the process of public engagement and its objectives. Regarding the question of *who*, respondents wanted to engage a variety of groups; “to bring school classes up in … those who are seventeen, eighteen or so, and have a debate” patients with disability, general public, parents, “statens offentliga utredningar … this sort of public investigations, special public investigations that the government is initiating,” members of parliament, and lastly, patient groups.

Public engagement was perceived as important because it avoids the following: letting one group solely influence healthcare decision-making, namely, the healthcare sector (legal expert); misconstruing government’s motives for implementing such a program considering past experiences with eugenics in Germany (physician 2); and to prepare the public with sound knowledge which “makes (them) more relaxed, (feel) more safe and can see the advantages with the technique” (physician 3); and lastly, politicians now are “very sensitive to current trends.” One participant declared

I think Sweden absolutely needs more of (public engagement). So Sweden has, I think, a poor tradition here. And I look to the UK, I like the way that they have these public interactions around different things … And they have a whole sort of system set up for that, so anyone can be like a commentator to a proposal, which I think is fantastic. And we have nothing like that here. Here it goes on, the proposal goes to agencies, some selected experts. Other ones have to make an effort to comment. And I think that’s a bad thing (bioethicist 2).

Despite the positive attitude towards public engagement some respondents questioned the extent of public engagement because

if we let the public decides then it is going to be the strong groups probably that get their will. It is going to be the highly educated, the people who are in a well socio-economic state and then we do not follow the ethical principles of everybody’s (equal) value and to give the one with the highest need, help first (physician 4).

This would give rise to “a problem of justice” (bioethicist 3). Moreover, the public is not equipped with necessary expertise to conduct “conceptual or ethical analysis” and do not rely on “principled reasoning” when they give their opinions (bioethicists 1 and 3). Thus, participants made a distinction between allowing the public to decide on a policy and permitting public debate on healthcare issues.

To answer the question *how*, respondents indicated several means to engage the public either via media, public lectures, or scientifically based questionnaires.

### Research

Three main areas of research were articulated by respondents in relation to preconception ECS, namely, *research on ethical issues*, *health economics research*, and *research on test panels.*

A few respondents indicated the necessity to conduct research and analysis of ethical issues potentially raised by preconception ECS, as well as evaluating the experience of ethics committees in other countries. Moreover, a respondent raised the notion of well-being: what it means and how preconception ECS can affect it.

For health economics research, respondents highlighted cost benefit analysis as one respondent explained;

I think you need to know the outcome, how many, if we use this (preconception ECS), how many disabilities would it be that we could somehow avoid. Because screening the public, that would be a humongous cost and how many more healthy children would it lead to (physician 4)

As for the test panel, half of the respondents requested studies to shed light on reliability, specificity and sensitivity of the tests, number of false positive and false negative results, and systematic review of the scientific evidence that currently exists. One respondent called for research to be conducted on “the condition itself. Is it going to be a real impairment on the future human being” (legal expert).

### Responsibility

Responsibility was a recurrent theme that surfaced during the interviews. Under this theme, three subthemes emerged: *paternal* and *societal* responsibility as well as the concept of *responsibilization*. By the later concept, one respondent explained

If you do tests like this to a larger extent then people perhaps to a larger degree will think that they have a responsibility to find out and it would be irresponsible not to find out, to opt out. And I suppose nobody has seriously thought that this should be compulsory in the sense that you would be fined or go to prison if you opt out, it would still be voluntary in that sense I think that most would be prepared to argue. And perhaps the voluntariness would be sort of not real, sort of illusory if there is this responsibilization that I need to find out, my neighbor found out, my sister found out, etcetera, so I can't be this irresponsible as regards the future health of my potential children (bioethicist 3).

Nevertheless, many interviewees held the view that there is no parental responsibility to undergo preconception ECS, presenting several reasons. One is preconception ECS should be regarded as an opportunity, not an obligation; another is “It seems a bit farfetched to think that you would find very many” positive cases and “In Sweden we have also decided that we shouldn’t, whether people make responsible decisions or not, should not be taken into account. That’s sort of part of the legal framework, in a sense” (bioethicist 1).

Societal responsibility surfaced as a subtheme when interviewee 2 stated

generally screening, when you are looking for disorders in people who are not sick, you have a very special responsibility as a society. It’s a very different situation compared to when people have symptoms and seek attention and you have to help them … It’s a very different situation from a responsibility point of view if the society goes out and looks for diseases. So you have to be extra careful about values like what you know about risks, what you know about benefits, what you know about the quality of the services that you provide (physician 1).

### Societal effects

Within this theme, the respondents reflected on the conception of society and potential effects of preconception ECS on it. These included *long-term effects* and *seeking after a perfect society.*

According to interviewees, Sweden is currently a disabled-friendly society.

We try to establish a society where you can live with a disability, quite a good life. We don’t have the resources to support everyone, but the vision is that … to be born with a disability well there you should be compensated for it and not to have the view in society, well, we have to abort because the society can’t help me to bring up this child (political party representative).

To a majority of respondents (eight respondents), programs such as preconception ECS reinforce notions associated with seeking a perfect society. Physician 4 stated

I hope we do not have a future where you decide who is going to give you the best... who are you going to get the best baby with and you base your relations on that and so on, so on. …..I do not know, baby factories is a bad word but … But if it (getting babies) is just going to be a production to society (physician 4).

Moreover, participant 4 worried that the spectrum of what is defined as normal is being narrowed down when we use such screening programs, because

To me there seems to be a tendency that we want to have perfect children. And I think there is a danger to that because they will be children … Or no one is perfect, really. So we're sort of closing in on the normality and I think this is a danger (legal expert).

And “we should give life only to perfect persons” (political party representative), which would result in elitism, as suggested by,

I mean, maybe you can know not only diseases in the future, you can get information on appearance, on intelligence or whatever and … it is also I think, create a sort of elite people I think. You want only the best one and the other ones are not allowed to live or … it is kind of an elite thinking, too much elite to only allow certain children to be born (physician 3).

Not everyone were of the same view on “perfection,” one respondent said,

I think it’s impossible to, in a sense, achieve perfect men, but still I think conditions that cause a lot of suffering, premature death … I think we have a better society without those conditions. I mean, we could argue we have tried to get rid of some contagious diseases and I think we are better off without them, so why … I can’t see any strong reasons why we wouldn’t have the same good reasons to get rid of cancer and heart and lung diseases and cystic fibrosis, etcetera, if we could. I mean … there will always be … even if there was not genetically, they would get mutations during the way and etcetera, etcetera, so … But if we can reduce mortality and morbidity in society, yes, I think that’s a good idea actually. Then we can, perhaps, focus on other stuff that are not perfect in society (bioethicist 1).

Another related notion that surfaced “varumärke” (trademark/branding) was perceived as a positive development in the society. This notion can be strengthened by preconception ECS.

We are more individualized, we think about branding concerning our own life … Branding … We think of ourselves as a varumärke. How we are, so to say, *uppfattade* (regarded). The picture of us, the common picture of us is very important as we act in social media, as we dress, as we ask people. It’s a part of the autonomy, that we look upon ourselves as strong persons, we make our own decisions and no other person will make them for us and decide our choices. It’s up to us … And also I think we are having some sort of empathy concerning others’ right to their own choices (theologian).

In addition, some interviewees were of the view that preconception ECS may decrease the value of life and human value. There is a risk to regard people with disease as “have(ing) a lesser value” (physician 2) and eventually,

It also puts intolerance to these people who do not fulfill this criterion if you should … you can do abortion for all these four hundred and ninety-nine conditions. That creates … I think it creates an intolerant society (physician 3).

Regarding long-term effects, respondents suggested a variety of potential outcomes. The most frequently stated is enhanced societal pressure to test or blame parents who decide against undergoing preconception ECS screening, particularly if it results in children with disability. This may be coupled with increased stigmatization of those born with disability. Moreover, finding a partner can become a challenge and some children may not be born because parents decide against giving birth to affected offspring. This, over the long run, may result in increased immigration as a result of lower birth rates.

One respondent elaborated in more detail of how the “structural social long-term consequences” can happen. The participant explained:

And here’s the question that when you give information to people, even if it doesn’t really benefit them, society will now view them as more responsible. Because they know more. So it’s their fault. And this is kind of a … almost automatic thing. This happens in the public mind because this is some very, very core beliefs that almost everybody has about the link between knowledge, freedom and responsibility … Then comes this structure dynamic risk that if the mindset of the population changes regarding how they view the responsibility of people then the willingness to change these laws might come, right. So it might be more and more that hey, why should I pay for their affected child, for instance (bioethicist 2).

One participant raised the issue of genetic exceptionalism as a possible effect by stating,

… if you do this population wide, screen all people when they’re eighteen or something,…… then you would have an enormous focus in the healthcare system and in the society about genetic susceptibility towards disease so it might increase the so-called genetic exceptionalism, that genetic information is so important in order to determine our future health … But as regards to common folk diseases like cardiovascular disease and high blood pressure and things that most people actually die from and suffer from genetic information is of very little practical relevance. And you might sort of increase an unsound focus on the genetic factors behind ill health. (bioethicist 3)

## Discussion

Policymaking experts, in our study, raised several social and ethical concerns pertaining to the possible implementation of preconception ECS in Sweden. Though participants were of varied professional backgrounds (bioethicists and non-bioethicists), they all exhibited competence in presenting and discussing ethical and social matters. This is in part not only because they have been serving on instituted governmental agencies or professional organizations’ boards engaged, primarily, with addressing such issues but also because there is general political commitment in Sweden to upkeep certain values as prescribed in the Swedish Healthcare Act SFS 2017:30 (Ministry of Social Affairs 2017). For example, the sole function of SMER, which has been assigned by the Swedish Government, is to respond to inquiries on ethical matters pertinent to biomedical technological advances (Socialdepartementet 2018. Moreover, the Commission on the Future of Sweden’s report of Strömbäck (2013) pinpointed that tackling ethical controversies, expected to surface with increased use of modern medical technologies, is one of the future challenges facing Sweden. This commitment is not confined to the government only, non-governmental professional organizations, such as Swedish Society of Medicine, have in its auspices ethics committees to partake in healthcare-related ethical debates in Sweden (Svenska Läkaresällskapet 2018; Sveriges Läkarförbund 2016).

Despite the apparent commitment of the government and non-government organizations to ethical and social matters, studies indicate that the practice is far from perfect. Garpenby and Nedlund (2016) reported on the backstage practices among Swedish politicians, who engaged in priority setting proceedings of a local health authority. According to their respondents, priority setting decisions are fast paced leading to convergence of decision-making to political leaders, who usually possess better comprehensive knowledge of the process. Consequently, it reinforced “elitist” structure of the proceedings and decisions (Garpenby and Nedlund 2016). This is reflected in the results in our study as well. In addition, it gave little time for politicians to ponder ethical issues adequately (Garpenby and Nedlund 2016). Höglund and Falkenström (2018) analyzed seven national and local healthcare policy documents for ethical values and conducted in-depth interviews of 13 elected and non-elected government officials to examine how ethical procedures were integrated in their work and decision-making. The documents showed sufficient evidence of reference to what participants in our study described as “Swedish values,” namely, human dignity, equality, solidarity, autonomy, and prioritization of resources. In practice, however, the officials stated there was low integration of ethics particularly in management decisions including financial ones. So, cost efficiency was concerned with finding the cheapest alternatives and ethical discussions were perceived as relevant at clinical settings only (Höglund and Falkenström 2018).

Yet economics, according to our respondents, is a pivotal matter in relation to preconception ECS as the overall cost of the screening program is predicted to be high for the society and the fear is that it would displace resources from more urgent areas of healthcare, such as cancer treatments. Prioritization and efficient use of resources, as well as finding alternative means of financing, are integral components of healthcare policymakers’ function. It can be argued that policymakers are responsible for distributive justice and equality in healthcare provision for a collective, in this case the Swedish society. This contrasts with the situation of healthcare professionals, whose main responsibility is treatment and care of patients irrespective of the therapy cost. Such distinction was presented by healthcare professionals in an earlier study by Matar et al. (2016).

Among the recommendations the WHO proposes for effective healthcare systems are utilization of evidence and/or research and public engagement to guide policymaking (Hanney et al. 2003). These requirements were reiterated by our policymakers in relation to ECS but the extent of public engagement was disputed among them. Policymaking should not in its entirety rely solely on input from the public but include the views of other stakeholders. The reasons given are as follows: the public lacks expertise in formal methodical ethical analysis; there is a risk of stronger societal groups hijacking the debate and the voice of the most vulnerable is lost. Though the attitude may seem paternalistic, some argued that Swedish policymakers’ responsibilities include ensuring equity and tending to the needs of the most vulnerable (referring to values incorporated in the healthcare act (Ministry of Social Affairs 2017)).

When respondents, in our study, advocated better public engagement, they referred collectively to both the patient groups and general public without making a distinction. In their article, Fredriksson and Tritter (2017) argued for differentiating between patient vs public engagement based on several reasons. In terms of roles and interests, patient organizations contribute with the point of view of a healthcare user and their experiential knowledge of their illness and thus address issues such as available treatments, privacy, consent, or right of access for a particular patient group. In contrast, the public attends to matters from a citizen’s point of view and discusses henceforth healthcare at a macrolevel, in short, as a public policy actor. So, collective concerns of welfare, rights, or responsibilities are of focal significance (Fredriksson and Tritter 2017). Again, the distinction between individual vs collective (or societal) theme emerges, here. The discussion is relevant for Swedish policymakers in order to define the objectives, and the type of public engagement as well as the extent it influences decision-making regarding preconception ECS.

It has been stated that evidence-based healthcare policymaking may incorporate empirical evidence in the form of research both quantitative and qualitative, as well as other forms of evidence, for instance, expert opinions and users’ accounts or experience (Brownson et al. 2009; Choi et al. 2005; McQueen 2001). In relation to preconception ECS, evidence is still accumulating because it is an emerging field (Benn et al. 2014; Ready et al. 2012; Wienke et al. 2014; Wilfond et al. 2018). The need for more research has been endorsed by professional organizations and providers (Edwards et al. 2015; Henneman et al. 2016; Lazarin and Haque 2016) and was reiterated by policymakers in our study. The research as required by respondents was investigating health economics aspects, social and ethical issues, and features of test panels, in respect to the Swedish milieu.

A similar study to investigate Dutch stakeholders’ needs and barriers to implementing ECS was conducted in the Netherlands. Among those interviewed were scientists/researchers, healthcare professionals, members of patient organizations, and policymakers. Similar to our results, stakeholders identified different interest groups, such as patient groups, professional organizations, and the public who would lobby for implementing ECS, each with a divergent interest in mind. The professional groups are advocating ECS implementation to enhance freedom of choice, whereas the public at the time did not feel an urgency to demand the test. Also, adjusting financial structures, as to ECS expenses and compensations of healthcare personnel, was voiced as means to guarantee equity of access. Organizing the infrastructure to accommodate implementation, need for research to investigate the long-term effects of mass screening, and provision of training and education to healthcare personnel and the public all conform to the responses communicated by our research participants. Lastly, assigning responsibility as to who takes the lead in the process, whether it be professionals, patient groups, or the public, was perceived as a need for implementing ECS (Holtkamp et al. 2017).

The concept of responsibility was raised in a different context, in our study, as respondents assigned no responsibility to parents to undergo screening. Preconception ECS was viewed as an opportunity to make reproductive decisions; and regardless of the choice or its result, parents should not be blamed for such a choice. In fact, there was an angst that implementing such programs will lead to responsibilization; in essence, a parent feels a responsibility to test because of his/her perception that the state/the society are entrusting him/her with such a responsibility when, in fact, they are not.

In its article on responsible implementation of ECS (2016), the European Society of Human Genetics (ESHG) put forth a list of recommendations for responsible use of ECS. There is a consensus that the main motivation for implementing ECS should be assisting voluntary-informed reproductive decision-making (Henneman et al. 2016), a motive that was questioned by policymakers in our study, as to whether taxpayers’ money should be used to achieve such a goal.

Among the societal effects discussed in relation to preconception ECS, in previous literature, are risks of stigmatization/discrimination of the disabled, eugenics, medicalization, and healthcare inequity (De Wert et al. 2012; Henneman et al. 2016; Scully 2008). In this study, respondents raised similar concerns and elaborated on societal long-term effects, for instance, a change of public mind-set from tolerance and societal responsibility for the disabled (the current status in Sweden) to intolerance and attitudes of blame, which in time could be translated into hostile laws against the disabled. Another notion raised is the possibility of confining the scope of normality and what is perceived as “normal” to a very narrow definition. All these potential outcomes were attempts to create a perfect society, where a healthcare system employs recent technologies to create perfect offspring that would cause minimal disruption of the system. Indeed, further “technification” of reproduction was made analogous to a process of industrial production of babies, with eventual change in public perception of human value and dignity.

Sweden, being part of the European Union, is affected by events and policies transpiring in other EU countries, especially if such policies become EU directives. These political considerations were in the mind of policymakers in our study. So, notions of free movement and access of tests in other countries, implementing local laws that do not cross borders, and pluralistic ethical outlooks of EU countries were political issues to be taken into account.

## Strengths and limitations

Expert interview, as a method of inquiry, is relatively novel and had been incorporated in political science research to examine political actions and their implementation and routine practices in politics. It is a method that aims to access experts’ knowledge and their decision-making methods (Abels and Behrens 2009). Consequently, this method seemed the best to capture the objectives of our study. Nevertheless, the literature indicates there are challenges encountered during the expert interview process, since the interaction could be affected by differing genders, “interests, trust, power, control, and hierarchy.” In terms of gender, it was noticed that female interviewers possessed better communication skills, were more open to feedback, and were deemed less intimidating compared to men. Moreover, women interviewing women, in male-dominated fields, showed more understanding and sisterhood attitudes (Abels and Behrens 2009). As stated earlier, the interviewees may perceive the interviewer as an expert and therefore may resort to counter-questioning or inquire about the interviewer’s standpoint on an issue (Bogner and Menz 2009).

To combat these partialities, we adopted a neutral stance during the interviews and respectfully avoided taking sides. With regard to gender influence, positive aspects, such as open communication, were maintained, while negative effects like eliciting biases or defensive attitude were minimized to the best of our ability. Also, it is recommended to select experts from different organizations to ensure better quality of data and minimize biases and we believe this has been accomplished in our study.

Our respondents have been selected because of their unique specialized knowledge and decision-making roles as well as their insight into the workings of health policymaking in their respective organizations. If preconception ECS is to be implemented in Sweden, most of, if not all, the committees to which our interviewees belong will be involved in the ethical and social deliberations. Thus, the method of expert interviewing, despite its shortfalls as discussed earlier, seems the best to answer the research questions of the study.

As stated previously, trustworthiness of analysis was given due consideration. To ascertain credibility, we included in our sample a variety of professions, genders, and ages, as well as different institutions that influence healthcare policymaking in Sweden. Moreover, a representative sample was coded by the last author and the generated themes were compared to the results of the first author. During two meetings, the authors discussed and explained their points of views and differences were reconciled. Furthermore, data saturation was reached and further interviewing generated no new ideas or concepts. The collection of data and the analysis process were carried out over a period of 1.5 year, with minimal alteration to the interview guide and therefore dependability was maintained. Lastly, transferability was best achieved via the use of interviewees’ quotes in the presentation of results.

The varying ages, sexes, professional background, and their affiliations are strong factors in enhancing trustworthiness of the study. However, as all qualitative studies, the results are not generalizable to all healthcare policymakers. Nevertheless, the study gave a profound insight into the workings of healthcare policymakers and their deliberations to evaluate a new HT.

Some of the interviewees were pressed for time yet they have responded to all the questions. Moreover, the interviews were conducted in English, which is a second language for the researchers and the respondents.

## Conclusions and future implications

In this study, preconception ECS is considered as a new HT which warrants ethical and social evaluation before making a decision to implement it as part of the Swedish healthcare system.

It can be concluded that, according to our respondents, Sweden is currently not ready to implement a preconception ECS as part of the healthcare system. This is due to several ethical and social concerns yielded by the study, which also include potential long-term effects. The main motivation for ECS, as recommended by ESHG, is facilitating informed reproductive decision-making, which respondents regarded as a dubious reason to spend taxpayers’ money on. In addition, respondents were afraid of potential long-term consequences of preconception ECS on Swedish values, such as prizing human dignity and allocating priority of care to the most vulnerable.

However, respondents acknowledged the different stakeholders and were open to engaging the public’s views in the policymaking process. This is a way to combat the current status of healthcare policymaking in Sweden, which is viewed to rely mainly on politicians, experts, and authoritative entities. Moreover, they recognized the potential influences of EU and worldwide healthcare policies on the Swedish ones.

Future implications encompass research with other Swedish stakeholders, such as parents or the general public to examine their views on preconception ECS.
